# Visual-somatosensory integration (VSI) as a novel marker of Alzheimer’s disease: A comprehensive overview of the VSI study

**DOI:** 10.3389/fnagi.2023.1125114

**Published:** 2023-03-30

**Authors:** Jeannette R. Mahoney, Helena M. Blumen, Pierfilippo De Sanctis, Roman Fleysher, Carolina Frankini, Alexandria Hoang, Matthew J. Hoptman, Runqiu Jin, Michael Lipton, Valerie Nunez, Lital Twizer, Naomi Uy, Ana Valdivia, Tanya Verghese, Cuiling Wang, Erica F. Weiss, Jessica Zwerling, Joe Verghese

**Affiliations:** ^1^Department of Neurology, Division of Cognitive and Motor Aging, Albert Einstein College of Medicine, Bronx, NY, United States; ^2^Department of Medicine, Division of Geriatrics, Albert Einstein College of Medicine, Bronx, NY, United States; ^3^Department of Pediatrics, Albert Einstein College of Medicine, Bronx, NY, United States; ^4^Gruss Magnetic Resonance Research Center, Albert Einstein College of Medicine, Bronx, NY, United States; ^5^Division of Clinical Research, Nathan S. Kline Institute for Psychiatric Research, Orangeburg, NY, United States; ^6^Department of Psychiatry, NYU Grossman School of Medicine, New York, NY, United States; ^7^Department of Radiology, Montefiore Medical Center, Albert Einstein College of Medicine, Bronx, NY, United States; ^8^The Dominick P. Purpura Department of Neuroscience, Albert Einstein College of Medicine, Bronx, NY, United States; ^9^Department of Psychiatry and Behavioral Sciences, Montefiore Medical Center, Albert Einstein College of Medicine, Bronx, NY, United States; ^10^Department of Radiology, Division of Nuclear Medicine, Montefiore Medical Center, Bronx, NY, United States; ^11^Department of Epidemiology and Population Health, Albert Einstein College of Medicine, Bronx, NY, United States; ^12^Center of Aging Brain, Montefiore Medical Center, Yonkers, NY, United States

**Keywords:** multisensory integration, sensory processing, mobility, cognition, Alzheimer’s disease

## Abstract

Identification of novel, non-invasive, non-cognitive based markers of Alzheimer’s disease (AD) and related dementias are a global priority. Growing evidence suggests that Alzheimer’s pathology manifests in sensory association areas well before appearing in neural regions involved in higher-order cognitive functions, such as memory. Previous investigations have not comprehensively examined the interplay of sensory, cognitive, and motor dysfunction with relation to AD progression. The ability to successfully integrate multisensory information across multiple sensory modalities is a vital aspect of everyday functioning and mobility. Our research suggests that multisensory integration, specifically visual-somatosensory integration (VSI), could be used as a novel marker for preclinical AD given previously reported associations with important motor (balance, gait, and falls) and cognitive (attention) outcomes in aging. While the adverse effect of dementia and cognitive impairment on the relationship between multisensory functioning and motor outcomes has been highlighted, the underlying functional and neuroanatomical networks are still unknown. In what follows we detail the protocol for our study, named The VSI Study, which is strategically designed to determine whether preclinical AD is associated with neural disruptions in subcortical and cortical areas that concurrently modulate multisensory, cognitive, and motor functions resulting in mobility decline. In this longitudinal observational study, a total of 208 community-dwelling older adults with and without preclinical AD will be recruited and monitored yearly. Our experimental design affords assessment of multisensory integration as a new behavioral marker for preclinical AD; identification of functional neural networks involved in the intersection of sensory, motor, and cognitive functioning; and determination of the impact of early AD on future mobility declines, including incident falls. Results of The VSI Study will guide future development of innovative multisensory-based interventions aimed at preventing disability and optimizing independence in pathological aging.

## Introduction

Alzheimer’s disease (AD) affects over 6 million Americans and is the most-common cause of dementia (Alzheimer’s Association, 2022). AD follows a prolonged, progressive disease course that begins with pathophysiological changes affecting individuals’ brains years before any clinical manifestations are observed (Jack et al., 2013). The notion that Alzheimer’s modifies sensory processing is in its very early stages (Albers et al., 2015). Yet, this supposition is supported by evidence demonstrating that amyloid-beta (Aβ) protein accumulates in sensory-association areas of the brain well before higher-order cognitive areas like the prefrontal cortex (PFC; Thal et al., 2002). While it is well known that mobility impairments are common in mild cognitive impairment and AD (Beauchet et al., 2008; Verghese et al., 2008a), the National Institute on Aging (NIA) has recognized that functional changes in sensory and motor systems also modulate the progression of AD. Thus, the NIA is supportive of new initiatives aimed at discovering novel, non-cognitive and non-invasive biomarkers for early detection of Alzheimer’s disease, and this is directly in line with the research priorities of our division.

There is a well-established association of higher-order cognitive processes including attention and executive functioning with balance (Woollacott and Shumway-Cook, 2002; Zettel-Watson et al., 2015), gait (Verghese et al., 2007b, 2008a; Holtzer et al., 2012; Groeger et al., 2022) and falls (Hausdorff and Yogev, 2006; Holtzer et al., 2007) in healthy, as well as cognitively impaired older adults. In fact, the PFC has been found to play a critical role in successful gait and cognition (Beauchet et al., 2016). Work from our division has linked gait to discrete brain structures such as cerebellar, precuneus, supplementary motor, insular, and PFC (Blumen et al., 2019). Additionally, we have found: (1) associations between walking performance and functional connectivity in sensory-motor and fronto-parietal resting-state networks (Yuan et al., 2015); (2) links between gray matter volume in areas involved in multisensory integration (including superior temporal sulcus and superior temporal gyrus) with aspects of gait and gait control (Tripathi et al., 2022); and (3) significant associations between gait and visual somatosensory integration (VSI) processes (Mahoney and Verghese, 2018, 2020). However, the interplay of multisensory, cognitive, and motor processes and the underlying functional neural networks involved remain largely undefined in healthy and pathological aging.

**FIGURE 1:**
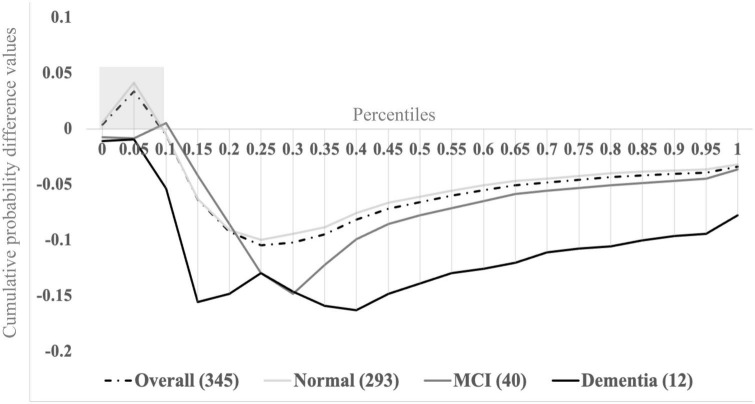
Visual-somatosensory integration (VSI) cumulative probability difference waves overall and by cognitive status (normal, mild cognitive impairment, or dementia). Adapted from Mahoney and Verghese (2022). Reprinted by permission of Oxford University Press on behalf of The Gerontological Society of America.

Sensory inputs emanating from a device like a cell phone (that simultaneously lights up, vibrates, and plays a ringtone) combine in the brain to yield faster responses than responses to individual unisensory components, thereby decreasing the time it takes to answer the phone. The *magnitude of multisensory integration* can be quantified using established probabilistic modeling procedures of behavioral performance, such as reaction time (RT) and accuracy (Mahoney and Verghese, 2019). *Magnitude of multisensory integration* is operationalized as the area-under-the-curve of the difference between actual and predicted cumulative probability distribution functions during a pre-identified portion of the difference waveform. For example, Figure 1 depicts cumulative probability difference values (*y*-axis) between actual and predicted distribution functions from our latest study for percentile binned RT responses ranging from 0.0 to 1.0 in 5% increments (Mahoney and Verghese, 2020).

The combined study cohort (*n* = 345; dashed trace) reveals successful multisensory integration processes (i.e., positive cumulative probability difference values) during the fastest tenth (0.0–0.1) of RTs. Here, the area under the curve during the 0.0–0.1 percentiles (gray shaded box) is operationalized as the *magnitude of multisensory integration* (a continuous measure). Higher values indicate superior ability to integrate visual-somatosensory information (i.e., benefit from multisensory inputs), whereas lower and negative values indicate inability to integrate or to benefit from multisensory inputs. Stratifying the overall group based on cognitive status assigned during consensus case conference procedures [normal cognition (*n* = 293) – solid light gray trace; mild cognitive impairment (MCI; *n* = 40) – solid dark gray trace; and dementia (*n* = 12) – solid black trace] revealed that *magnitude of multisensory integration* is significantly reduced for individuals with MCI or dementia. Further, cognitive status significantly mediated the relationship between *magnitude of multisensory integration* and measures of mobility, such that older adults with cognitive impairments demonstrated impaired multisensory integration and significantly slower gait, as well as poorer balance compared to older adults without cognitive impairments (Mahoney and Verghese, 2020). Our findings further revealed that VSI is also correlated with attention-based performance measures (Mahoney et al., 2012; Mahoney and Verghese, 2020) that may target PFC regions known to be compromised in AD. Consequently, we argue that multisensory integration has potential utility in early AD detection, though further work is needed to uncover the exact structural and functional neural correlates of VSI.

### Significance

Balance requires efficient interactions between musculoskeletal and sensory systems (Shumway-Cook and Woollacott, 2012), which are compromised in aging (Lord et al., 2007). Poor balance is a major predictor of falls, a leading cause of injury and death in older Americans. Our research reveals that better *magnitude of VS integration*, is associated with better balance and gait, as well as decreased risk of falls (Mahoney et al., 2019). Our previous investigations, however, did not determine the association of impaired VSI with early dementia stages, nor its contribution to mobility decline.

Impairments in cognition could adversely affect the association between *magnitude of multisensory integration* and mobility measures because: (1) multisensory processing appears to be regulated by PFC (Jones and Powell, 1970; Cao et al., 2019); (2) selective attention modulates multisensory integration in aging (Hugenschmidt et al., 2009; Mozolic et al., 2012); and (3) disruptions in executive attention and cognition in aging compromise multisensory integration and mobility processes (Yogev-Seligmann et al., 2008; Holtzer et al., 2012; Mahoney and Verghese, 2020). Although our preliminary findings are encouraging and of high public health significance, we believe that we are only scratching the surface for a much-needed larger multisensory investigation. The proposed study, from here on referred to as The VSI Study, is significant as it will identify the functional neural correlates of VSI, while also determining whether Alzheimer pathology concurrently impacts sensory integration and motor processes. The goal of The VSI Study is to determine the combined influence of multisensory, cognitive and motor changes in early Alzheimer’s disease in an effort to shape the development of future innovative multisensory-based interventions, prognostic tools, and new research-driven therapies aimed at preventing disability and optimizing independence in pathological aging.

### Specific aims

**FIGURE 2:**
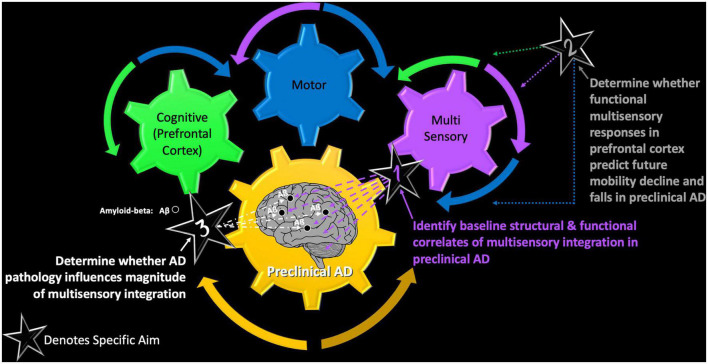
The VSI Study – a conceptual model of the main objectives of the VSI Study and how each afford examination of the intersection of cognitive, motor, and multisensory functioning in healthy and pathological aging.

The VSI Study seeks to achieve three main specific aims denoted as stars in Figure 2. In this conceptual model, cognitive, motor, and (multi) sensory functioning are depicted as individual gears that must work together to transmit a behavioral response. However, the impact of preclinical AD on each of the individual gears, as well as on the overall system (requiring successful interactions across all functions) requires systematic examination. Thus, our three main study aims are as follows:

#### Identify baseline structural and functional neural correlates of VSI in preclinical AD

Results from The VSI Study employing multimodal neuroimaging procedures will provide a deeper understanding of the structural and functional neural correlates of VSI in older adults with normal and preclinical AD. Here, preclinical AD will be defined as manifesting impaired cognitive performance [performance worse than 1.5 standard deviations from the mean on standardized neuropsychological tests] and presence of elevated Aß in plasma at baseline using established cut scores (Bateman et al., 2019). We hypothesize that the *magnitude of VSI* will be correlated with gray matter volume, cortical thickness, and blood-oxygen-level-dependent (BOLD) signal activation in *subcortical* and *cortical* regions of interest including dorsal lateral prefrontal cortex (DLPFC), rostral middle frontal, and superior frontal gyrus at study baseline (Year 1). We predict that older adults with preclinical AD will manifest reduced *magnitude of VSI* (worse), decreased cortical volumetrics, decreased functional connectivity, and lower BOLD responses when compared to older adults with normal cognition.

#### Determine whether VSI task-related BOLD activation in prefrontal cortex predicts future mobility decline and falls

Individuals with Alzheimer’s disease are at greater risks for falls and mobility disability, but specific causes of AD and the temporal onset of functional changes across systems are currently not known. We have shown a mediating effect of dementia and mild cognitive impairment on the relationship between VSI and motor outcomes (Mahoney and Verghese, 2018; Mahoney et al., 2019). These results suggested that individuals with cognitive impairments manifested poor VSI and poor balance/slow gait. Using an fMRI task where participants are asked to respond as quickly as possible to unisensory visual, unisensory somatosensory and combined visual-somatosensory stimuli in a 3-Tesla (3T) magnet, our second specific aim will determine whether visual-somatosensory task-related BOLD activation in the prefrontal cortex at baseline predicts future mobility (gait) declines and risk of incident falls. We hypothesize that preclinical AD causes disruptions in subcortical and cortical (multisensory, motor, and cognitive) regions that modulate multisensory, motor, and cognitive functions necessary for efficient mobility.

#### Assess the validity of VSI as a novel Alzheimer’s behavioral marker

The validity of VSI as a novel marker for AD will be established by correlating the *magnitude of VSI* with presence of Aβ using plasma-based measures at baseline. In Year 2, positron emission tomography (PET) measures affording localization of Aβ deposits (Piramal Imaging) to estimate Aβ neuritic plaque density will also be examined in relation to *magnitude of VSI*. Aβ protein deposition has been documented in both sensory and cognitive areas (Thal et al., 2002; Jack et al., 2018, 2019; Bateman et al., 2019). Therefore, we hypothesize that increased Aβ accumulation in sensory and cognitive areas, areas related to increased AD pathology, will be associated with decreased *magnitude of VSI*.

In keeping with the NIA-AA research framework (Jack et al., 2018), our innovative and timely project will distinguish AD symptomology (presence of mild cognitive impairment) from AD pathology (Aβ accumulation), while also applying the AT (N) classification system [Aβ (A), tau (T), and neurodegeneration (N)] to attain more direct assessment of neuropathologic changes. More specifically, and in keeping with the goals of establishing whether *magnitude of VSI* is a novel and early biomarker of AD, associations of VSI with plasma-based total and phosphorylated Tau, neurofilament (NfL), ApoE, and multimodal neuroimaging measures of Neurodegeneration will also be examined for study completeness.

### Innovation

Multisensory integration is not well-understood in aging and its relation to cognitive and motor functioning is recognized as a major knowledge gap in the field (Meyer and Noppeney, 2011; Wallace, 2012; Mahoney and Barnett-Cowan, 2019; Campos et al., 2022). The NIA recognizes that functional changes in sensory and motor (i.e., non-cognitive) systems have an impact on the development and progression of AD and requests identification of novel, non-cognitive non-invasive predictors to aid in early AD detection. Our multisensory integration research meets this request, while also addressing the knowledge gap and providing significant public health implications. We are recognized as the first group to have established the clinical utility of *magnitude of VSI* in aging by linking it to poor motor outcomes including loss of balance, falls, and gait decline.

Additional innovation highlights of the VSI Study include: (1) access to established research infrastructure and existing collaborations; (2) cost and time-efficient design affording access to *a priori* identified participants with and without preclinical AD; (3) longitudinal design affording comprehensive examination of systemic changes (and their interactions) over time on the progression of AD and its subsequent link to mobility declines; (4) novel project with comprehensive multimodal neuroimaging approach providing clear clinical application of results; and (5) identification of a novel, non-cognitive behavioral marker that simultaneously taps multiple integrative systems that have not been systematically examined in previous AD investigations.

The current study also provides innovation beyond its specific aims as it affords: (1) a deeper investigation of the onset of functional systemic changes over time; (2) comprehensive investigation of the neurobiological consequences of AD and its links to medical co-morbidities in relation to VSI processes given previously reported diminished multisensory integration in older adults with diabetes (Mahoney et al., 2021); (3) enhancement of multisensory digital health tools like CatchU^®^ used to screen and prevent falls for older adults in clinical settings (Mahoney et al., 2022); and (4) development of future multisensory-based interventions that will further enhance quality of life for seniors.

## Methods

### Study design

We propose a longitudinal study of older adults with (*n* = 104) and without (*n* = 104) preclinical AD; participants meeting criteria for dementia or AD will be excluded. In accordance with the NIA-AA research framework (Jack et al., 2018) and as stated earlier, our innovative project will allow us to disentangle differences in outcomes related to Alzheimer’s symptomatology [mere presence of mild cognitive impairment syndrome at established clinical case conference (Holtzer et al., 2008)] from those related to Alzheimer’s pathology (Aβ accumulation). Based on our previous studies (Mahoney and Verghese, 2020), we expect our preclinical AD group will include older adults with varying levels of cognitive impairment, ranging from amnestic and mixed MCI to preclinical AD. Sub-groupings of MCI and mild stage AD will afford *post hoc* analyses aimed at examining the impact of cognitive impairment syndromes on multisensory integration processes.

**FIGURE 3:**
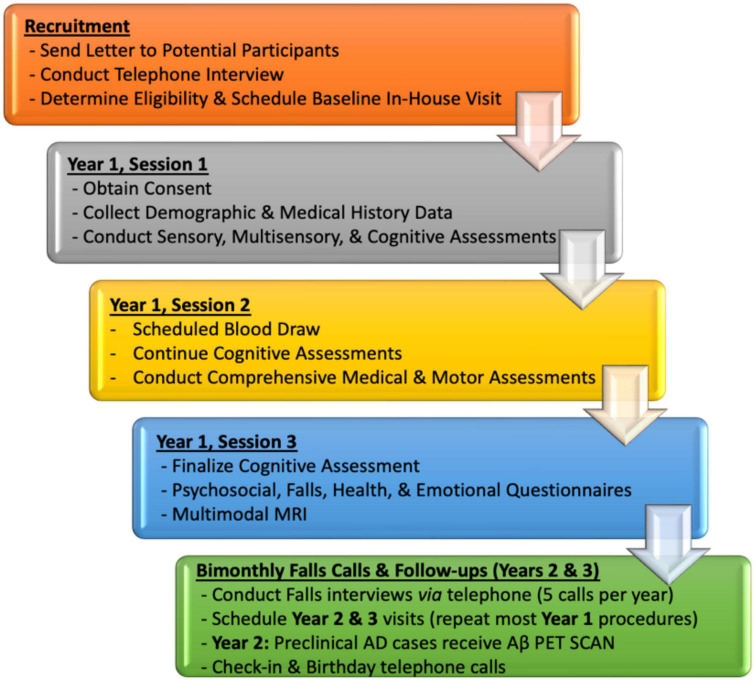
VSI Study flow by Year with high-level overview of targeted domains by baseline (Year 1) and follow-up sessions.

Interested participants will undergo extensive neuropsychological, sensory, physical functioning (mobility), neuroimaging, and blood testing, though we recognize that participants may decline participation in some procedures. The VSI Study includes three study sessions in Year 1 with subsequent follow-up calls every 2 months (to monitor falls) and yearly in-house visits in study Years 2 and 3. Initial enrollment of all 208 participants will be staggered across study Years 1–3, with follow-up visits conducted during study Years 2–5. Baseline sessions, designed using established divisional research studies as a model, aim to minimize fatigue and maximize effort by spreading test procedures out over three study sessions, each lasting about 3–4 h in duration (see Figure 3 for overview of study procedures by session). Based on our experience with previous and currently NIH-funded divisional studies that have similar protocols, we estimate a 90% completion rate for this protocol.

### Recruitment and study criteria

Participant recruitment for this project will be strategic. We will utilize existing infrastructure, recruitment methods, and available registration lists over 600 eligible and interested participants from previously funded divisional studies (R01AG036921, R01AG044007, and 1R01AG050448; and K01AG049813) for enrollment in the VSI Study. Adults aged 65 and older living in the NY metropolitan area may also be contacted using a commercially available third-party list. We have used these and other lists to recruit over 1,000 participants for various aging studies over the past 12 years. Identification of older adults with preclinical Alzheimer’s disease will be supplemented by clinical recommendations from neurologists and neuropsychologists (Drs. Verghese, Weiss, and Zwerling), as well as clinical patient lists from Montefiore’s Center of Excellence for Alzheimer’s disease (CEAD), including both the Center for the Aging Brain (CAB) and the Memory Disorders Center. Since the VSI Study builds on existing research infrastructure, we will ensure similar distributions of age, gender, and ethnicity for older adults with and without preclinical Alzheimer’s disease by monitoring demographic and clinical parameters and adjusting as needed as we accrue our sample.

In terms of our recruitment procedures, we will first mail letters to participants explaining the VSI Study. Then, the research team will follow-up by telephone and inquire whether the letter was received. If the participant received the letter, the research assistant will conduct standardized telephone recruitment interview procedures. If a letter was not received, the participant’s name and mailing address will be verified by the research team and a new letter will be mailed out, which will be followed by a telephone interview call. Interested participants meeting study eligibility criteria (see Table 1 for detailed inclusion and exclusion criteria) will be scheduled to come to the Albert Einstein College of Medicine, Division of Cognitive and Motor Aging, Sensorimotor Integration in Aging Lab for all in-house study sessions. After baseline study procedures are completed, the VSI Study case consensus team will convene and provide clinical diagnoses based on neuropsychological performance, neurological exam, medical history, and Aβ plasma results. Study group assignment will be determined during Year 1 case-conferences, and monitored every study year.

**TABLE 1:** VSI Study eligibility criteria.

A	General inclusion criteria
1	Adults aged 65 and older, residing in New York Metropolitan area who plan to be in area for next three or more years.
2	Able to speak English at a level sufficient to undergo our cognitive assessment battery.
3	Ambulatory. Participants are classified as “non-ambulatory” if they are unable to leave the confines of their home and attend a clinic visit. Participants who require walking aids to walk outside but are able to complete our mobility protocols without an assistive device or the assistance of another person will not be excluded.
**B**	**General exclusion criteria (one or more criteria)**
1	Presence of dementia [Telephone based Memory Impairment Screen score (T-MIS) of < 5, Alzheimer’s disease 8 (AD8) ≥ 2, or dementia diagnosed by study clinician at initial visit].
2	Serious chronic or acute illness such as cancer (late stage, metastatic, or on active treatment), chronic pulmonary disease on ventilator or continuous oxygen therapy or active liver disease. Individuals with recent cardiovascular or cerebrovascular event (MI, PTCA, CABG, or stroke) will not be excluded if they meet above inclusion criteria.
3	Mobility limitations solely due to musculoskeletal limitation or pain (e.g., severe osteoarthritis) that prevent participants from completing mobility tests. Mere presence of disease will not be used to exclude participants if they can complete the mobility tasks.
4	Any medical condition or chronic medication use (e.g., neuroleptics) in the judgment of the screening clinician that will compromise safety or affect cognitive functioning or terminal illness with life expectancy less than 12 months.
5	Progressive, degenerative neurologic disease (e.g., Parkinson’s disease or ALS) diagnosed by study clinician and as per medical history.
6	Presence of clinical disorders that overtly alter attention like delirium.
7	Hospitalized in the past 6 months for severe illness or surgery that specifically affects mobility (e.g., hip or knee replacement) and that prevent participants from completing mobility tests or plans for surgery affecting mobility in the next 6 months.
8	Severe auditory, visual, or somatosensory impairments: Vision is screened using a Snellen chart – significant loss of vision is defined as corrected vision less than 20/400 on the Snellen chart with both eyes. Hearing is initially evaluated as part of the screening telephone interview. Participants will be excluded only if they are unable to follow questions asked in a loud voice during in-house sessions. Somatosensory functioning will be measured using quantitative sensory threshold protocols and presence of neuropathy will be assessed using the Michigan Neuropathy Screening Instrument.
9	Active psychoses or psychiatric symptoms (such as agitation) noted during the clinic visit that will prevent completion of study protocols. Past history of these symptoms or presence of psychiatric illness not used as exclusion criteria.
10	Living in nursing home.
11	Participation in intervention trial. Participants can participate in other observational studies.

### Study measures

A comprehensive list of established assessment measures is delineated in Table 2 by domain and session. The variables of interest and their applications will be explained in detail below as they relate to each specific aim. Note that additional measures (i.e., pilot measures) unrelated to the study’s specific aims may be included in the protocol but are not listed here. Variables and test measures labeled in green will be used as covariates in certain statistical models, depending on the specific research aim. As noted earlier, our central hypothesis is that preclinical AD is associated with neural disruptions in subcortical and cortical areas that concurrently modulate sensory, motor, and cognitive functions, resulting in mobility decline. Therefore, our study strategically includes a wide array of test measures in each domain.

**TABLE 2:** Assessment measures by domain and session.

Domain	Session	Assessment measures
Screening	Telephone Interview	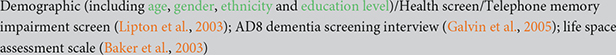
Sensory	Years 1–3	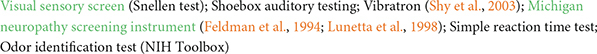
Multisensory	Years 1–3	Visual-somatosensory integration test (Mahoney et al., 2019); CatchU^®^ (Mahoney and Verghese, 2022)
Neuropsychological	Years 1–3	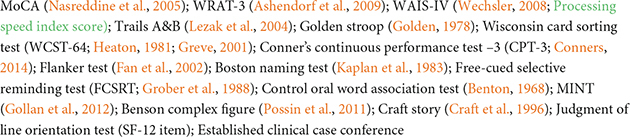
Neurological/Medical History/Physiological/Other	Years 1–3	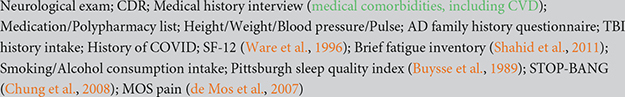
Gait/Mobility	Years 1–3	Quantitative gait assessment (Verghese et al., 2002b,2007a,^2009^); Normal pace walking/Walking while talking protocol (Holtzer et al., 2011) and Primary gait screen (Protokinetics); General mobility questionnaire
Balance/Physical Performance/Leisure	Years 1–3	Unipedal stance test (Hurvitz et al., 2000, 2001); Berg balance test (Berg et al., 1992); Biodex sensory organization test; ABC scale (Powell and Myers, 1995); Short physical performance test (SPPB; Guralnik et al., 1994); Stair climbing; Grip strength (Guralnik et al., 1994); Functional reach (Duncan et al., 1990); Purdue pegboard (Tiffin and Asher, 1948); Maze (Sanders et al., 2008); Leisure scale (Verghese et al., 2003)
Falls	Years 1–3	Baseline and bimonthly fall interviews (Verghese et al., 2004; Verghese et al., 2008b,^2009^); Falls self-efficacy scale (Tinetti et al., 1990)
Activities of Daily Living		4 ADLs; Instrumental ADLs (Lawton and Brody, 1970); Bathing scale
Psychosocial/Personality	Years 1–3	Geriatric depression scale (Brink et al., 1982); Beck anxiety inventory (Beck et al., 1988); Big-5 inventory (Barrick and Mount, 1991)
Social Support/Loneliness	Years 1–3	Social network index; MOS social support survey (Sherbourne and Stewart, 1991); UCLA loneliness index-3 (Russell, 1996).
Multimodal Neuroimaging	Year 1	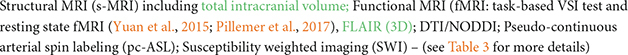
Blood and Plasma	Year 1	Basic chemistry; Lipid panel; Glucose/A1C; IL-6; CRP; Aβ/ApoE/pTau/Neurofilament (Bateman et al., 2019)
Amyloid Imaging	Year 2*	Fluorine-18 florbetaben (Neuraceq) PET scan in individuals with preclinical AD [*case confirmed→ Aß + plasma test and confirmed poor neuropsychological performance]

Established study procedures labeled in 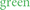 for use as covariate in statistical models.

Our independent variable is *magnitude of VSI* derived from our established VSI test (Mahoney and Verghese, 2019), and our dependent variables include neuroimaging measures of functional integrity (BOLD signal and resting state functional connectivity), motor outcomes (balance, gait, and falls), and Alzheimer pathology (Aß presence and accumulation). Comprehensive screening measures, neuropsychological and neurological/medical history assessments will be used to ensure study appropriateness, characterize our cohort, as well as aid in determination of cognitive status and study group enrollment. Additional psychosocial, social, emotional, and personality measures are included for study completeness as they will foster future research initiatives.

### Primary research outcomes and statistical plan by aim

As stated earlier, our group has linked the *magnitude of VSI* to important cognitive and motor outcomes (Mahoney et al., 2014; Mahoney and Verghese, 2018, 2020; Mahoney et al., 2019). Furthermore, we highlighted the adverse effect of dementia and mild cognitive impairments on these outcomes (Mahoney and Verghese, 2020). However, the functional neural substrates of VSI have not been identified in healthy or cognitively impaired adults. The justification for identifying associated functional neural networks of multisensory integration will allow us to design novel multisensory-based interventions to complement existing interventions that demonstrate some fall reduction. The VSI Study will employ a theoretical and empirical approach to determine whether VSI is indeed a novel non-cognitive, non-invasive predictor of early Alzheimer’s disease and specifically, address the following research aims:

#### Identify baseline structural and functional neural correlates of VSI in preclinical AD

Participants will complete a simple reaction time (RT) test employing three bilaterally presented conditions (visual, somatosensory, and multisensory visual-somatosensory) and a control (i.e., “catch”) condition where no stimulation is presented, and no response is expected. The four stimulus conditions will be randomly presented with equal frequency (15 trials per condition per block, 3 blocks, yielding a total of 180 trials). The addition of “catch” trials and variable inter-stimulus-interval (ranging from 1–3 s) impedes anticipatory effects (see Mahoney and Verghese, 2018; Mahoney and Verghese, 2019, 2020; Mahoney et al., 2019; for details). Participants will be instructed to respond to all stimuli as quickly as possible. Performance accuracy will be defined as the number of accurate stimulus detections divided by 45 trials per condition. Using our established methodology, robust probability (P) models that compare the cumulative distribution function (CDF) of combined unisensory visual (V) and unisensory somatosensory (S) reaction times with an upper limit of 1 min [P (RT_*V*_ ≤ *t*) + P (RT_*S*_ ≤ *t*), 1] to the CDF of multisensory visual-somatosensory (VS) reaction times [P (RT_*VS*_ ≤ t)] will be implemented. For any latency *t*, the inequality holds when the CDF of the actual multisensory VS condition [P (RT_*VS*_ ≤ *t*)] is less than or equal to the predicted CDF {min [P (RT_*V*_ ≤ *t*) + P (RT_*S*_ ≤ *t*), 1]}. When the actual CDF is greater than the predicted CDF (i.e., positive value), the model is violated, and the RT facilitation is the result of multisensory interactions that allow signals from redundant information to integrate or combine non-linearly. Predicted CDF will be subtracted from the actual CDF to form a difference curve. *The area-under-the-curve of the group-level violated portion of the difference curve will serve as the continuous measure of magnitude of VSI.*

All neuroimaging procedures will be conducted at the Gruss Magnetic Resonance Research (MRRC) Center at Albert Einstein College of Medicine under the direction of Dr. Lipton. The MRRC offers state-of-the-art multimodal neuroimaging on Philips whole-body Ingenia Elition 3.0 Tesla Magnetic Resonance Imaging (MRI) scanner equipped with 32-channel head coil. Multimodal MRIs will be captured at baseline (i.e., study Year 1) and processed by our neuroimaging team consisting of Drs. Blumen, Fleysher, and Hoptman. Our non-invasive multimodal MRI imaging techniques are reliable and have been used extensively in both healthy aging and dementia studies in our division/department. For the VSI Study, specific MRI outcome measures are listed by modality in Table 3. Structural MRI (sMRI; ∼5 min) will be acquired using high-resolution T1-weighted whole head structural imaging using axial 3D-MP-RAGE acquisition over a 240 mm field of view (FOV) with 1.0 mm isotropic resolution. TE = 4.6 ms, TR = 9.9 ms, α = 8°, and SENSE factor = 2.6 (left-right) x 2 (head-foot). Functional MRI (∼10 min total) will be acquired using whole brain T2* weighted images with echo planar weighted images with echo planar imaging over a 224 mm FOV on a 112 × 112 acquisition matrix, 3 mm slice thickness (no gap); TE = 30 ms, TR = 2,000 ms, flip angle = 90°, SENSE factor = 2 and 42 trans-axial slices per volume. The fMRI procedures will measure BOLD activation (outcome measure) during the VSI task. This event-related design emulates our established psychophysical protocol where 60 trials of visual alone, somatosensory alone, multisensory visual-somatosensory (VS; Mahoney and Verghese, 2019) will be presented in the scanner, but will also include the above-mentioned “catch” trials (60 trials). The visual (V) stimulus will be bilateral asterisks presented for 100 ms on a VisuaStim digital visor (Resonance Technology, Inc., Northridge, CA, USA). The somatosensory (S) stimulus will be bilateral pneumatic pulses presented for 100 ms through the Somatosensory Stimulus Generator system (4-D Neuroimaging) which is compatible in the MRI scanner. These stimuli will be presented alone and concurrently in the case of the concurrent VS stimulus. The critical contrast here will examine differences in BOLD activation between the multisensory VS condition *vs*. the sum of the two unisensory conditions (V + S). Resting-state (rs)-fMRI (10 min) will also be captured while participant lay still and relax (i.e., a passive no-task condition) with their eyes open. Fluid Attenuated Inversion Recovery (FLAIR; ∼5 min total) will account for white matter hyperintensities (WHI) indicative of small vessel disease. FLAIR will be acquired using whole head imaging sagittal 3D-TSE-IR acquisition over a 250 mm FOV with 1 mm isotropic resolution. TE = 338 ms, TR = 4,800 ms, TI = 1,650 ms TSE Factor = 182, compressed SENSE acceleration factor 3.5. FLAIR results will account for presence/absence of small vessel disease and will be considered as a covariate. Additional multimodal neuroimaging procedures to be included for study completeness, beyond the scope of the specific aims include: Susceptibility Weighting Imaging (SWI); Pseudo-Continuous Arterial spin labeling (pc-ASL); and Neurite orientation and dispersion density imaging (NODDI) – see Table 3 for details.

**TABLE 3:** List of Magnetic Resonance Imaging (MRI) procedures.

MRI measures	Modality	Outcome measure(s)
Structural	Structural MRI	Cortical thickness and gray matter volume
	3D FLAIR	Presence of white matter hyperintensities and lacunes
	Susceptibility Weighting Imaging (SWI)	Presence of microbleeds
	Pseudo-continuous arterial spin labeling (pc-ASL)	Quantitatively measures tissue perfusion, or cerebral blood flow (CBF)
	Neurite orientation and dispersion density imaging (NODDI)	A diffusion imaging technique to detect cortical and corticospinal tract neurodegeneration (N)
Functional	Functional MRI (fMRI)	BOLD response (beta) during VSI task
	Resting state fMRI	Fisher *z*-transformed Resting state functional connectivity

In terms of our statistical approach for Aim 1, the *magnitude of VSI* (independent variable) will be analyzed and quantified using established probabilistic modeling procedures (Mahoney and Verghese, 2019). The dependent measures of structural and functional neural integrity include: (1) cortical thickness: (2) volumetric measures for regions of interest extracted from structural MRI; (3) Beta-weights for the multisensory contrasts for each region of interest extracted from task-based fMRI; and (4) Fisher *z*-transformed resting-state functional connectivities between pairs of regions extracted from resting-state fMRI.

Covariates identified in our prior studies (Holtzer et al., 2007; Verghese et al., 2007b,2008a; Mahoney and Verghese, 2018, 2019, 2020), including but not limited to age, gender, ethnicity, medical comorbidities (including cardiovascular disease), total intracranial volume, and attentional capacity will be selected to account for their influence on VSI and association with outcomes. Participants will be categorized into two groups based on preclinical AD diagnosis at baseline. All statistical approaches will be supervised by our study statistician, Dr. Wang.

SAS 9.4 (Cary, NC) will be used for the analyses. We will conduct multivariate mixed effects models for imaging outcomes for the following *a priori* selected regions of interest including: dorsal lateral PFC, rostral middle frontal, and superior frontal gyrus regions, superior temporal sulcus, motor cortex, thalamus, basal ganglia, hippocampal, and cerebellum (one per outcome, with group factors as necessary). These regions are selected based on preliminary findings in 100 older adults (*unpublished data*) which reveal significant (*p* < 0.05) associations between magnitude of VSI and measures of structural integrity (defined here as either volume or cortical thickness) in the following regions: parahippocampal (memory); caudal middle-frontal dorsal lateral prefrontal cortex (DLPFC: cognitive functions - especially executive & attention); superior temporal sulcus (STS; multisensory); precentral (motor), postcentral (somatosensory), and lateral occipital (visual). Preliminary fMRI findings in 56 healthy older adults (ages 65–92; *unpublished data*) further supports inclusion of these regions given significant associations between VSI magnitude and blood-oxygen-level-dependent (BOLD) responses in known multisensory (middle temporal), motor (basal ganglia), and cognitive areas (PFC including DLPFC). The outcomes in these models include measures of neural integrity, structural (volume and thickness), and/or functional (BOLD) activation isolated by region, and the predictor of interest is the *magnitude of VSI*. Additional models will further include cognitive status (normal or preclinical AD) and its interaction with *magnitude of VSI*. The effect of cognitive status (normal or preclinical AD) on *magnitude of VSI* will be evaluated using similar mixed effects models. The hypothesis-driven analyses will be limited to BOLD activation in the aforementioned regions of interest. Models will be run unadjusted and then adjusted for confounders.

#### Determine whether VSI task-related BOLD activation in prefrontal cortex predicts future mobility decline and falls in preclinical AD

Here, we propose that reduced VSI activation in specific regions of PFC (fMRI BOLD responses), will be associated with worse balance (unipedal stance), slower gait (worse Pace scores), and increased risk of incident falls. Additionally, we propose that preclinical AD will reduce VSI activation in PFC regions of interest, which will in turn adversely affect mobility measures. Our longitudinal design will allow us to identify the impact of functional changes in (multi)sensory, motor, and cognitive processes (and their interactions) on the progression of AD that result in mobility decline.

The VSI task and multimodal neuroimaging procedures for this aim have been described above in Aim 1. For Aim 2, the VSI task will be run in the magnet to obtain fMRI task-related BOLD responses with concurrent psychophysical data. The following aim-related mobility procedures [established tests that have been validated and utilized in our center for over two decades (Verghese et al., 2002a,2007b,2008a,^2009^, ^2012^; Holtzer et al., 2007, 2012, 2014; Verghese and Xue, 2010, 2011; Ayers and Verghese, 2014; Mahoney et al., 2014, 2017, Holtzer et al., 2014; Mahoney and Verghese, 2018, 2020)] will be included in Aim 2:

Balance (∼2 min) will be assessed using unipedal stance time, which requires individuals to balance their body weight with foot on the ground for a maximum of 30 s (Hurvitz et al., 2000, 2001). Unipedal stance time is a widely used clinical test that is listed under NIH’s toolbox. Poor scores on this test have been associated with presence of neuropathy (Hurvitz et al., 2001), and predict falls in older adults (Hurvitz et al., 2000). This test will be administered twice during each study visit and maximum unipedal stance time (sec) will serve as the outcome measure.

Quantitative Gait (∼5 min) will be assessed on a 28-foot instrumented walkway (PKMAS system; Zenometrics LLC) with embedded pressure sensors that provides spatial and temporal gait parameters including: gait velocity, stride length, percentage of double support, stride time, stance time, cadence, stride length variability, and swing time variability. Participants will be assessed twice while walking on the mat at their everyday pace. Gait velocity, as well as the Pace Factor score comprised of gait velocity, stride length and percentage of double support, will serve as dependent measures.

History of falls (∼5 min) in the past 1 year, number of incident falls over a 3 year longitudinal study-period, and fall information such as type, injury and location will be tracked at yearly in-house interviews and during bimonthly telephone interviews using established criteria and standardized questionnaires (Tinetti et al., 1994). Falls are defined as sudden, unintentional, unprovoked changes in body posture, not due to a major intrinsic event (stroke) or overwhelming hazard. Dichotomous ratings of fall-history over the past 1 year (0, 1), presence of incident fall over study period (0, 1) and time to fall/censor will serve as outcome measures.

The association of functional VSI activation in specific PFC regions of interest with mobility measures of balance and quantitative gait will be examined cross-sectionally at baseline (Year 1), using linear regression models. Linear mixed effects models (LMEM) will be used to examine the association of baseline functional VSI activation in the PFC on the changes in the longitudinal balance and quantitative gait performance. The predictors will be examined as continuous variables to facilitate clinical translation of results. Adjustments for multiple comparisons will be made. Additional LMEMs will be employed to examine interplay and time course of multisensory, cognitive, and motor functioning. Cox proportional hazard model will be used to evaluate the association of *magnitude of VSI* with the risk of incident falls (Mahoney et al., 2019) and hazard ratios (HR) with 95% confidence intervals (CI) will be reported. Time to fall will be recorded as number of days from baseline study date to the interview date when the fall was recorded. If the participant does not report a fall, the follow-up time will be defined as the number of days from the baseline in-house visit to the last date of contact. Repeated incident falls will be examined using Andersen-Gil extension of Cox model (Anderson and Gill, 1982) and Poisson models. Robust sandwich covariance estimates account for correlations among multiple events within the same participant. Cox models will be adjusted for potential confounders. Proportional hazards assumptions of all models will be tested graphically and analytically. We will also apply mediation analysis using product of coefficients methods to evaluate whether cognitive status (normal vs. preclinical AD; independent variable) causes variation in PFC-related VSI activation (mediator), which in turn causes variation in specific mobility measures (dependent variables) using separate mediation models (balance and pace). Mediation analyses will be run using IBM’s Statistical Package for the Social Sciences (SPSS-28) and Hayes’ PROCESS package (Hayes, 2018). Confidence intervals that do not include 0 for the mediator will be defined as mediation.

#### Assess the validity of VSI as a novel Alzheimer’s behavioral marker

Alzheimer’s disease is associated with build-up of specific proteins (i.e., biological markers) in the brain, namely Amyloid-βeta (Aβ) in the form of plaques (Thal et al., 2002) and tau (T) in the form of neurofibrillary tangles. Common brain imaging techniques such as MRI or Computerized Tomography (CT) do not afford assessment of amyloid plaques and neurofibrillary tangles. However, molecular imaging procedures like Positron Emission Tomography (PET) imaging directly visualize these characteristic features of Alzheimer’s disease. Aβ accumulates in sensory association areas well before higher-order cognitive areas like the PFC (Thal et al., 2002). In Aim 3, we predict that AD pathology (i.e., accumulation of Aβ) will be associated with decreased *magnitude of VSI* in preclinical Alzheimer’s disease participants. Here, presence of Aβ will be measured in blood at baseline using plasma-based assays and established cut-scores (Bateman et al., 2019). Individuals that are Aβ+ on plasma-based tests at baseline, will receive amyloid PET imaging in study year 2. The combined use of conventional MRI with these techniques will also contribute to the early identification of Alzheimer’s disease.

The experimental design for this aim has been described above. Beyond the VSI task the following specific Aβ procedures will be implemented to determine its association with *magnitude of VSI*, and ultimately its use as a novel and early biomarker for preclinical AD.

Plasma-Based Blood Testing will be conducted during baseline visits for each participant. Aβ (40 and 42) and Apolipoprotein E (ApoE) will be assessed by C_2_N Diagnostics lab using novel multiplexed assays (Jack et al., 2018). PrecivityAD™ accuracy for determining amyloid positive versus negative status was 86%. Blood samples will be collected and placed in Einstein’s biorepository. Frozen samples will be subsequently shipped to C_2_N Diagnostics to be processed. Results from plasma-based testing conducted on bloods drawn at baseline, in conjunction with neuropsychological performance, will be critical for study group assignment, as well as disentangling AD symptomology from AD pathology.

Amyloid PET Imaging will only be conducted in study Year 2 for participants enrolled in the preclinical AD group (*n* = 104). All PET scans will be conducted at Montefiore Medical Center by Dr. Valdivia and her team. Positron emission tomography (PET) is expensive and involves the use of an imaging device (scanner) and a radiotracer that is injected into the patient’s bloodstream. The radiotracer used to estimate Aβ neuritic plaque density for this specific aim is called Neuraceq (florbetaben F-18) and is manufactured by Piramal Imaging. PET imaging after the radiotracer is injected, will afford quantification of the distribution of Aβ in the brain, where affected brain regions containing Aβ will be tabulated.

The association of *magnitude of VSI* with presence of Aβ protein levels in both plasma-based Aβ levels (continuous measure) and amyloid PET scans (dichotomous ± rating by brain region) will be examined using logistic and linear regression models, respectively, while adjusting for potential confounders.

## Discussion

In summary, we propose to recruit 208 community-dwelling older adults with and without preclinical AD for a three-year longitudinal study. Our central hypothesis is that preclinical Alzheimer’s disease is associated with neural disruptions in subcortical and cortical areas that concurrently modulate sensory, cognitive, and motor functions, resulting in mobility decline. Our project seeks to address a NIH-identified high-priority research topic, where the interplay of multisensory integration with cognitive and mobility outcomes will be extensively studied in individuals with and without preclinical Alzheimer’s disease. A deeper understanding of the underlying neural correlates of VSI and their association with cognitive and motor outcomes will support the advance of novel, non-invasive, and non-cognitive AD markers, as well as foster the development of novel multisensory interventions designed to target specific neural derailments, while significantly augmenting existing interventions to prevent disability and optimize independence.

As with any study, there are potential pitfalls and limitations that should be discussed, along with strategies to mitigate any potential shortcomings. Missing data is a concern of any longitudinal study; to reduce the likelihood of missing data, we will reschedule study visits that are missed and update participant contact information annually. Though not a main objective, diffusion tensor imaging data will be collected and analyzed using FSL software to provide a measure of functional anisotropy. Further examination of multimodal neuroimaging relationships will be computed using probabilistic tractography between regions of interest (ROIs). Neuroimaging data access can enable investigations of additional cortical pathways not identified in our proposed neural circuit; our approach here, however, is to focus on theory-based predictions. We recognize that biomarkers, including the AT (N) classification system [Aβ (A), tau (T), and neurodegeneration (N)] recently developed the NIA-AA task-force, affords a more direct assessment of neuropathologic changes (Jack et al., 2019). In an effort to determine whether magnitude of VSI is a novel and early biomarker of mild stage Alzheimer’s disease, the current study will also assess plasma-based total and phosphorylated Tau, neurofilament (NfL) and (ApoE).

In line with current preventative approaches, results from The VSI Study will provide insight into the neurobiology of early AD and aid the development of novel prognostic tools and therapeutic interventions. The primary focus of this project will guide strategic design of new multisensory-based interventions for non-cognitive outcomes like falls. Although not a specific aim of the current study, development of future multisensory-based interventions in high-risk patients requires identification of the structural and functional neural networks involved in multisensory integration processes, as well as understanding of the impact of early AD on these networks and systems. Such knowledge will be essential to designing future remediation trials that target key PFC and other regions involved in multisensory integration to induce neural plasticity that will be associated with improvements in sensory, cognitive, and mobility outcomes for older adults with and without pre-existing cognitive impairments.

## Ethics statement

The studies involving human participants were reviewed and approved by the Institutional Review Board at the Albert Einstein College of Medicine. All participants provided written informed consent to participate in this study.

## Author contributions

JRM wrote the manuscript. All authors reviewed, revised, and approved the final manuscript before submission.
